# MAPbI_3_ Incorporated with Carboxyl Group Chelated Titania for Planar Perovskite Solar Cells in Low-Temperature Process

**DOI:** 10.3390/nano9060908

**Published:** 2019-06-23

**Authors:** Pei-Shan Li, Rathinam Balamurugan, Bo-Tau Liu, Rong-Ho Lee, Hsueh-Tao Chou

**Affiliations:** 1 Department of Chemical and Materials Engineering, National Yunlin University of Science and Technology, Yunlin 64002, Taiwan; m10315008@yuntech.edu.tw (P.-S.L.); balar@yuntech.edu.tw (R.B.); chouht@yuntech.edu.tw (H.-T.C.); 2 Department of Chemical Engineering, National Chung Hsing University, Taichung 40227, Taiwan

**Keywords:** perovskite solar cell, reactive crystalline TiO_2_, carboxyl group, MAPbI_3_

## Abstract

Low-temperature, solution-processed, highly efficient hybrid organic/inorganic perovskite planar heterojunction solar cells were fabricated by incorporating reactive crystalline titania (h-TAc) into MAPbI_3_ layers. The h-TAc was prepared by the sol-gel reaction at low temperature followed by solvothermal treatment. The photoelectrical properties of the solar cells with h-TAc were analyzed. The incorporation with 0.85-wt% h-TAc showed the highest power conversion efficiency (PCE, 15.9%), increasing 69% compared to the pristine cell. The enhancement arose from large-grained microstructures, leading to a low rate of charge recombination. The carboxyl groups chelated on the surface of h-TAc revealed a strong attraction to lead ions, which are significantly helpful to MAPbI_3_ crystal growth.

## 1. Introduction

Owing to their promising advantages such as low exciton binding energy, high charge carrier mobility [1,2,3,4,5,6,7], high absorption coefficient [8,9], and large charge carrier diffusion length [2,10,11], hybrid organic/inorganic perovskites have recently emerged as outstanding materials in photovoltaic technology [12,13,14]. Hence, the development of perovskite solar cells (PVSCs) with high power conversion efficiency (PCE) and cost-effectiveness is a challenging task that has become a very popular research area in the past few years [15,16,17,18,19,20,21,22,23,24,25,26,27]. The maximum PCE of PVSCs in 2019 reached 24.2%, as confirmed by the National Renewable Energy Laboratory [28]. In general, the quality of perovskite active layer with respect to morphology and crystallization strongly affects the performance of the PVSCs [1,29,30,31]. Significant progress has been made in the development of perovskite thin film quality, especially in preparation methods such as sequential deposition [18,32,33], dual source evaporation [29,34], solvent engineering [17], additive enhancement [35,36,37] and vapor-assisted solution processing [34] to achieve a high PCE. However, constructing highly crystallized perovskite thin film without defects is the key to obtaining a typical perovskite solar cell with satisfactory photovoltaic properties. 

A well-defined grain structure, full surface coverage, and small surface roughness are essential aspects for high quality perovskite layers. Some efforts have been made to develop a new processing method for the preparation of crystalline perovskite thin films such as fast deposition—crystallization in which extra solvent has been dripped on the film during the high speed spin-coating process [38], solvent annealing by using *N*,*N*-dimethylformamide (DMF) [39], rapid crystallization by solvent-solvent extraction [40], and induced crystallization by room-temperature mixed solvent vapor annealing [1]. Among these methods, solvent annealing and mixed solvent vapor annealing methods resulted in high PCE, indicating that solvent plays an important role in producing highly crystalline perovskite layer.

In our recent reports [41,42], highly reactive anatase TiO_2_ chelated by acetic acid (called as TAc) and the TAc treated by the solvothermal method (called as h-TAc) were incorporated into mesoporous TiO_2_ photoanodes in dye-sensitized solar cells (DSSCs), resulted in improved PCE. It was proven that the TAc/h-TAc increases the dye adsorption and decreases the charge transfer resistance, thereby improving the efficiency of DSSCs. In this study, we incorporated h-TAc into the perovskite active layer to improve significantly the PCE of the PVSCs. The h-TAc did not work as a mesoporous scaffold but reduced the electron/hole recombination in the perovskite layer. We have analyzed the effect of h-TAc on PVSCs and investigated the potential mechanism for h-TAc enhancement.

## 2. Experimental

### 2.1. Materials

Indium Tin Oxide (ITO)-coated glass substrates (7 Ω sq^−1^) and Spiro-OMeTAD were purchased from Ruilong Inc. (Taiwan). PbI_2_, lithium-bis(trifluoromethyl-sulphonyl) imide (Li-TFSI) and dimethyl sulfoxide (DMSO) were purchased from Sigma-Aldrich (St. Louis, MO, USA). Hydriodic acid and 4-*tert*-butylpyridine (tBP) were purchased from Alfa Aesar (Shanghai, China). Methylamine and PbI_2_ (99%) were purchased from ACROS chemicals (Fukuoka, Japan). P25, ST-01 and 18NR-T were obtained commercially from Evonik (Essen, Germany), Ishihara Sangyou (Osaka, Japan) and Dyesol (Queanbeyan, Australia), respectively. All chemicals were used as received. CH_3_N_3_H_3_I was synthesized by using the reported procedures [38] and the synthetic procedures were given in the Supporting Information.

### 2.2. MAPbI_3_ Solution

The h-TAc was prepared by the sol-gel method [43], followed by the solvothermal method, as reported previously [42] (see Supporting Information). CH_3_NH_3_I (0.1616 g) and PbI_2_ (0.467 g) were mixed in DMF and stirred for one day, resulting in a yellow solution of MAPbI_3_. As-prepared MAPbI_3_ solution (1 g) was mixed with various h-TAc contents (0, 0.75, 0.85 and 1.00 wt%). For comparison, MAPbI_3_ solution with other TiO_2_ of 0.85 wt% (P25, ST-01 or 18NR-T, respectively) was also prepared in the same manner.

### 2.3. Device Fabrication

Typically, the ITO glass was etched with zinc powder and diluted HCl (6 M), then cleaned with detergent, ethanol and acetone. ZnO solution was synthesized by hydrolysis and condensation of zinc acetate dehydrate (>98%, ACS) by potassium hydroxide in methanol using a Zn^2+^:OH^−^ ratio of 1:1.72 according to the reported procedure [44,45]. The ZnO solution was deposited on the etched ITO glass and spin-coated for 30 s at 3000 rpm followed by annealing at 100 °C for 10 min. The coating was repeated for a total of two layers to complete a dense layer. Forty L of MAPbI_3_ solution was dropped on the ZnO-coated glass and then spin-coated for 30 s at 5000 rpm in a nitrogen-purged glove box during the end of last 5 s; 105 L of toluene was dropped as anti-solvent and then annealed at 105 °C for 5 min to form a dark brown and glossy active layer. Spiro-OMeTAD (80 mg), 4-*tert*-butylpyridine (28.5 μL), and A 520 mg mL^−1^ Li-TFSI (17.5 μL) in acetronitrile were mixed in 1 mL of chlorobenzene. Afterwards, 20 μL of the solution was added dropwise on the MAPbI_3_ layer at 2000 rpm for 30 s. Finally, a Ag electrode was thermally deposited on the device through a shadow mask to define the effective active area of the devices (0.04 cm^2^).

### 2.4. Measurements and Characterization

The particle size distribution of the h-TAc was determined using a dynamic light scattering analyzer (NanoPlus, Micromeritics). The crystalline phase of the h-TAc and MAPbI_3_ was characterized through X-ray diffraction (XRD) using an X-ray diffractometer (Miniflex II, Rigaku) and Cu K radiation. The morphology of the device was examined using a field-emission scanning electron microscope (JSM-6700F, JEOL). The photocurrent density-voltage (J-V) characteristics were measured under irradiation of 100 mW cm^−2^ using a solar simulator (MFS-PV, Hong-Ming Technology) equipped with a source meter (Keithley 2400, Keithley Instruments). Electrochemical impedance spectra (EIS) were measured over the frequency range of 50–100 kHz with a potential perturbation of 10 mV using an electrochemical workstation (Zennium, Zahner). The absorption and emission spectra of ITO glass coated with ZnO and MAPbI_3_ were determined using a UV-Vis spectrometer (V770, Jasco) and a fluorescence spectrometer (LS-55, Perkin Elmer), respectively. Photoluminescence (PL) spectra of MAPbI_3_ layers were measured using fluorescence spectrophotometer (LS-55/45, PerkinElmer). The surface modification of TiO_2_ was examined using a Fourier transform infrared (FTIR) spectrometer (Spectrum One, PerkinElmer). For lead absorption analysis, 6 mg of TiO_2_ (h-TAc, P25, ST-01 and 18NR-T) were coated onto the glass followed by annealing at 100 °C for 1 h. Then, the TiO_2_ samples were immersed in a solution of PbI_2_ in DMF (10 mg/mL). After 5 min of lead adsorption, the samples were dried at room temperature for 10 min. Then, the sample was immersed in nitric acid about 10 min to desorb the lead ions. The lead ions were detected from the solutions for different kinds of TiO_2_ using an inductively coupled plasma-optical emission spectrometer (ICP-OES, OPtiMA 5100DV, Perkim Elmer).

## 3. Results and Discussion

**Figure 1:**
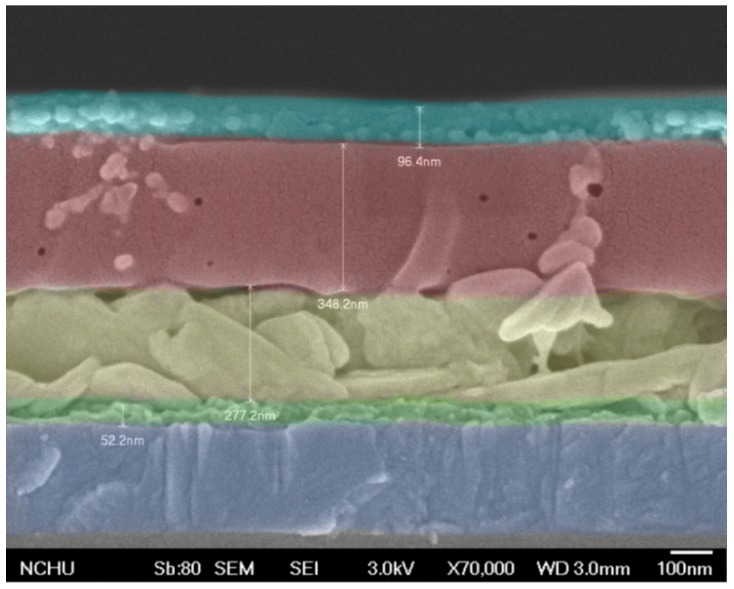
Cross-sectional SEM image of a typical perovskite cell fabricated in this study.

**Figure 2:**
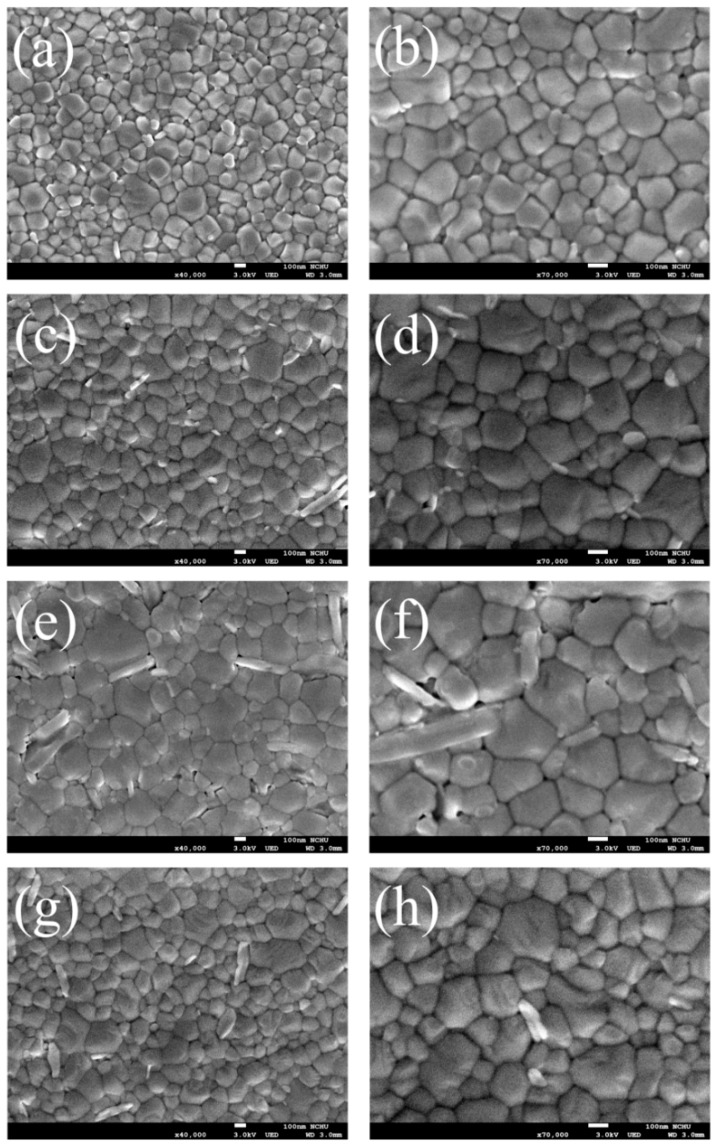
SEM images of MAPbI_3_ layer incorporated with various h-TAc contents: (**a**,**b**) 0, (**c**,**d**) 75, (**e**,**f**) 85 and (**g**,**h**) 100 wt%. Scale bar: 100 nm.

**Figure 3:**
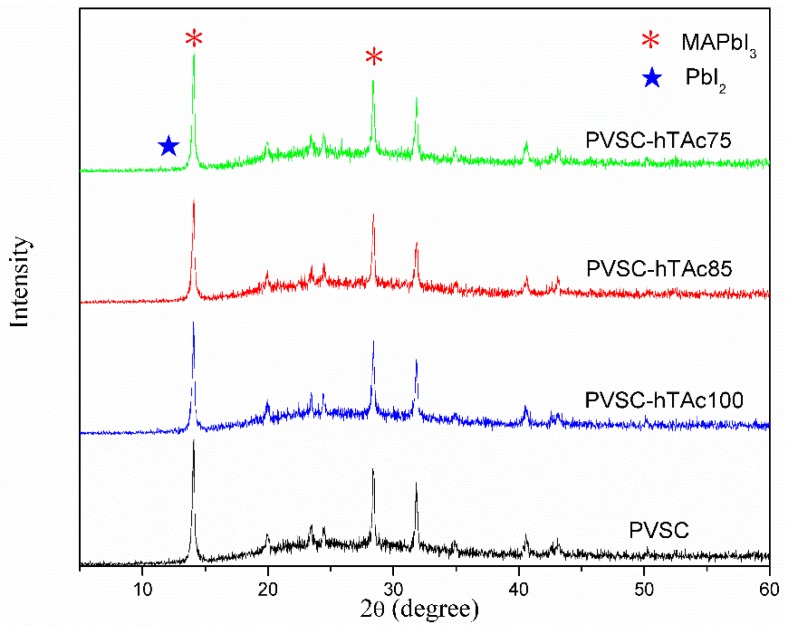
XRD patterns of MAPbI_3_ layer incorporated with various h-TAc contents.

The h-TAc, reactive anatase TiO_2_, was first synthesized in a low-temperature process by the sol-gel method and then put into an autoclave to improve its crystallinity. The particle-size distribution of the as-prepared h-TAc was over 55–115 nm, with an average diameter of 82.3 nm (Figure S1, Table S1). The h-TAc was incorporated into the active layer of conventional perovskite solar cells. The structure of a typical perovskite cell fabricated in this study is shown in Figure 1. The thicknesses of the layers for ZnO, MAPbI_3_, Spiro OMeTAD and evaporated Ag were approximately 52, 277, 348 and 96 nm, respectively. Observing in the cross-sectional image, the perovskite layer incorporated with h-TAc layer was sufficiently compact and dense. Figure 2 shows the SEM images of the morphology of the MAPbI_3_ layer with various h-TAc contents. The average crystal sizes of MAPbI_3_ with 0, 0.75, 0.85 and 1.00-wt% h-TAc incorporation, calculated form Figure 2, were 223.2 ± 42.9, 264.9 ± 58.1, 293.5 ± 34.6 and 243.8 ± 53.1 nm, respectively. Regardless of the amount of h-TAc in the perovskite layer, the crystal size became larger compared to the original one. 0.85-wt% h-TAc incorporation leads to a maximum crystal size. The XRD patterns of the perovskite layers with various h-TAc contents exhibited two peaks at 14.1° and 28.4° (Figure 3), corresponding to planes (110) and (220), respectively, of the perovskite structure of MAPbI_3_ [46]. No peak near 12.7°, attributed to PbI_2_ [47], was found on the curves. This result implies that h-TAc incorporation neither hinders the formation of crystal nor results in PbI_2_ separated out from MAPbI_3_.

**Figure 4:**
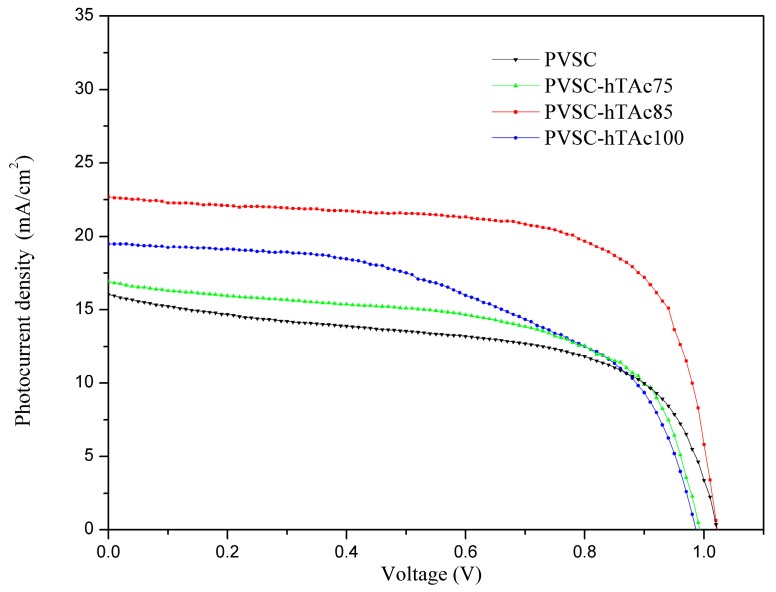
Photocurrent density-voltage curves of PVSCs incorporating various h-TAc amounts in the active layer.

**Figure 5:**
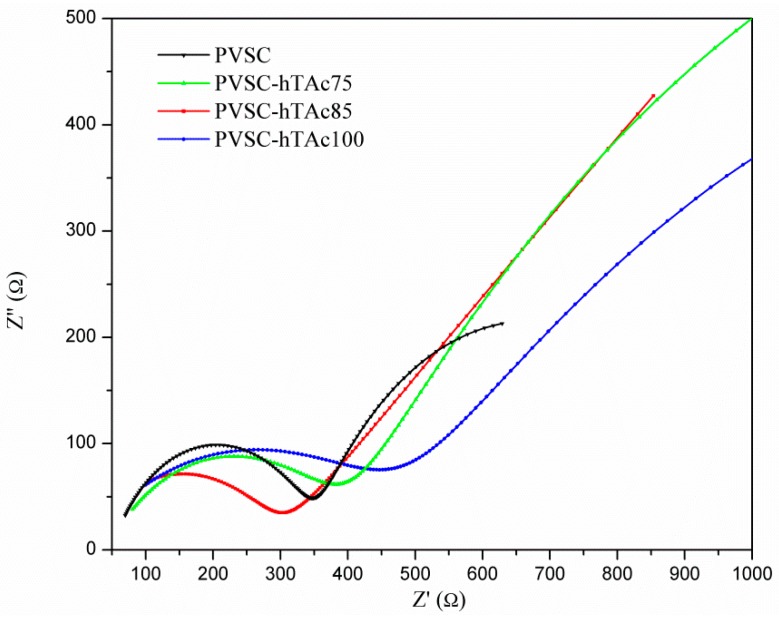
Nyquist plots of the electrochemical impedance spectra of PVSCs incorporating various h-TAc amounts in the active layer.

The J-V curves of PVSCs with/without h-TAc incorporation are shown in Figure 4. The corresponding characteristic properties are summarized in Table 1. It was found that the PCEs of the cells were increased with increasing h-TAc content, reaching a maximum at 0.85-wt% h-TAc incorporation (PVSC-hTAc85), and decreased with further increases of h-TAc content. The PCE can be enhanced maximally to 69% (from 9.46% to 15.9%) due to the h-TAc incorporation. Observed in Table 1, the enhancement on PCE is mainly attributed to the increase of photocurrent density. Figure S2 shows PL spectra of MAPbI_3_ with various h-TAc contents under 380-nm excitation. The PL intensity decreased with increasing h-TAc content, but increased with further h-TAc increase. The order follows that of a reciprocal of the PCE. Among the PVSCs, the PVSC-hTAc85 exhibited the lowest PL intensity but the highest PCE. Since fluorescence quenching indicates a decrease of electron-hole recombination [48], the enhancement of PCE caused by the h-TAc incorporation may have resulted from the suppression of charge recombination. Figure 5 shows Nyquist plots of the EIS for the PVSCs. The resistances appearing sequentially from high to low frequency are external (R1), interface (R2) and charge recombination (R3), respectively, summarized in Table 1. With the increase of h-TAc content, R2 increased slightly, whereas R3 increased significantly. The result indicates that the h-TAc incorporation increases the interface resistance but lowers the rate of electron-hole recombination, which is consistent with the observation on PL analysis. According to SEM analysis (Figure 2), the suppression of charge recombination may be ascribed to the increase of crystal size. However, too much h-TAc incorporation and a decrease in crystal size result in a decrease of charge recombination resistance (PVSC-hTAc100). The decrease of the charge recombination rate did not lead to increasing Voc [49], which might result from the coverage increase of perovskite film to inhibit the penetration of hole-transport-material solution [50]. In general, TiO_2_ particles are coated on the cathode as a mesoporous layer for PVSC fabrication [51,52,53,54,55,56,57,58,59,60,61]. In order to further realize the function of h-TAc on PVSCs, we also fabricated PVSCs with h-TAc as a mesoporous layer (PVSC-meso) instead of incorporating h-TAc into the perovskite layer. The fluorescent suppression was not observed for the PVSC-meso (Figure S3). Moreover, photoelectric properties were not improved using h-TAc as a mesoporous layer (Figure S4 and Table 2). The results indicate that the PCE of PVSCs is enhanced by h-TAc only when h-TAc is incorporated into the perovskite layer but not the mesoporous layer.

**Figure 6:**
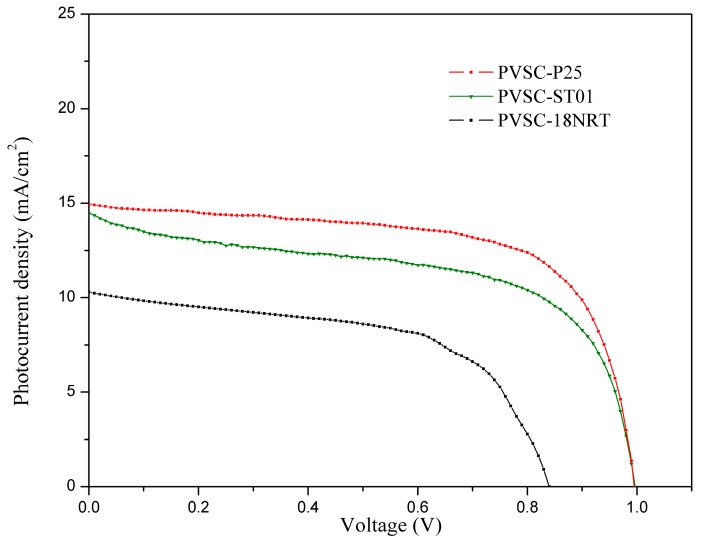
Photocurrent density-voltage curves of PVSCs incorporating various kinds of TiO_2_ in the active layer.

**Figure 7:**
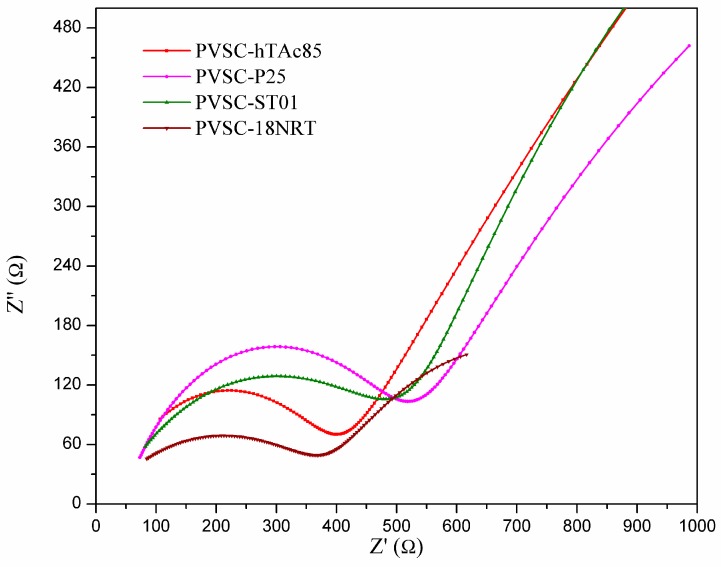
Nyquist plots of the electrochemical impedance spectra of PVSCs incorporating various kinds of TiO_2_ in the active layer.

The TiO_2_ incorporation into the perovskite layer was further investigated by replacing h-TAc with different TiO_2_ (P25, ST01 and 18NR-T). The surface morphologies of different TiO_2_-incorporated perovskites layers were studied by SEM analysis (Figure S5). It was observed that the average crystal sizes for P25, ST-01 and 18NR-T were 254.2 ± 66.3, 213.9 ± 40.0 and 215.6 ± 37.5, respectively. These crystal sizes were far smaller than those for h-Tac-incorporated PVSCs. Figure S6 shows the PL spectra for the PVSCs with different TiO_2_ incorporations. The h-Tac-incorporated PVSCs exhibited the lowest emission, whereas 18NR-T-incorporated PVSCs had the highest one. Indeed, the PVSC-hTAc85 revealed superior PCE compared with the other TiO_2_-incorporated PVSCs (Figure 6 and Table 3). The PVSC-18NRT had very poor photoelectric properties and worse PCE than PVSC-ST01, although both of them had similar perovskite crystal sizes. This result may be because of the formation of void defects in the 18NR-T-incorporated layer (Figure S7) due to solvent problems in slurry. Except for the PVSC-18NRT, all of the TiO_2_-incorporated PVSCs had close values for the open-circuit voltages (V_OC_), indicating TiO_2_ incorporation does not affect Voc. However, the values of J_SC_ were significantly different; the J_SC_ of PVSC-hTAc85 was nearly two times as high as those of PVSC-P25 and PVSC-ST01. The Nyquist plot and the corresponding EIS analysis for the TiO_2_-incorporated PVSCs are shown in Figure 7 and Table 3, respectively. Compared with other TiO_2_-incorporated PVSCs, h-TAc-incorporated PVSC had the highest charge combination resistance, which suppressed the recombination of separated electrons and holes. This result led to a large charge current and thus high PCE. We analyzed the adsorption of lead ions on the various kinds of TiO_2_ through inductively coupled plasma optical emission spectroscopy ICP-OES measurement, as shown in Table 4. The h-TAc showed extraordinarily high adsorption of lead ions (51.2 ppm) compared to other TiO_2_. The crystalline h-TAc possessed carboxylic groups bonded with Bronsted acid sites of titania (Figure S8) [42]. Because the carboxylic groups carried by h-TAc may effectively adsorb lead ions, h-TAc nanoparticles can work seeds or chelating agents to stabilize the growth of perovskite crystals, resulting in larger crystal sizes, lower rates of charge recombination and therefore higher PCE. The effect is similar to the capping used to stabilize perovskite crystals reported in the literature [62]. Although further analysis has not been done, the characteristics for stabilizing the perovskite layer by incorporating h-TAc are in favor of large-scale fabrication.

## 4. Conclusions

We reported a simple and effective strategy that enabled significant improvement in the PCE of PVSCs by incorporating h-TAc to perovskite layers. The h-TAc incorporation increased the MAPbI_3_ crystal size and also suppressed the electron-hole recombination. Incorporation of 0.85-wt% h-TAc revealed a maximum improvement, being a 69% enhancement over the PCE. No obvious enhancement on the PCE was observed when the h-TAc was used as the mesoporous layer or replaced by other TiO_2_ to be incorporated into the perovskite layer. The carboxyl group chelated on h-TAc can effectively adsorb lead ions, leading to increasing crystal growth of MAPbI_3_, thereby resulting in reducing the rate of charge recombination and increasing the PCE.

## Figures and Tables

**Table 1:** Photovoltaic characteristics of PVSCs incorporating various h-TAc amounts in the active layer.

Samples	h-TAc Content, wt%	Average Crystal Size, nm	Voc, V	Jsc, mA cm^−2^	FF, %	η, %	R_1_, Ω	R_2_, Ω	R_3_, Ω
PVSC	0	223.2 ± 42.9	1.02	16.0	57.8	9.5	50.0	301.6	645.6
PVSC-hTAc75	0.75	264.9 ± 58.1	0.99	16.9	60.0	10.0	41.0	355.1	2689.0
PVSC-hTAc85	0.85	293.5 ± 34.6	1.02	22.69	68.6	15.9	17.0	379.1	6143.0
PVSC-hTAc100	1.00	243.8 ± 53.4	0.99	19.5	52.6	10.0	1.7	483.7	2284.0

**Table 2:** Photovoltaic characteristics of PVSCs using h-TAc as the mesoporous layer.

Samples	the Mesoporous Layer	Voc, V	Jsc, mA cm^−2^	FF, %	η, %	R_1_, Ω	R_2_, Ω	R_3_, Ω
PVSC-meso	h-TAc	1.00	13.5	53.3	7.2	37.0	127.7	1793.0

**Table 3:** Photovoltaic characteristics of PVSCs incorporating different TiO_2_ into the active layer (0.85-wt%).

Samples	Kinds of TiO_2_	Average Crystal Size, nm	Voc, V	Jsc, mA cm^−2^	FF, %	η, %	R_1_, Ω	R_2_, Ω	R_3_, Ω
PVSC-P25	P25	254.2 ± 66.3	0.97	15.5	68.8	10.4	44.9	468.0	2741.0
PVSC-ST01	ST-01	213.9 ± 40.0	0.97	15.4	67.2	10.1	22.0	517.0	2566.0
PVSC-18NRT	18NR-T	215.6 ± 37.5	0.86	10.3	56.5	5.0	2.2	404.6	590.1

**Table 4:** Absorption of lead ions on different TiO_2_ determined by ICP-OES analysis.

TiO_2_ Samples	h-TAc	P25	ST-01	18NR-T
Lead ion, ppm	51.22	7.17	6.96	7.15

## Supplementary Materials

The following are available online at https://www.mdpi.com/2079-4991/9/6/908/s1, Synthesis of CH_3_NH_3_I, Preparation of h-TAc, Table S1: Particle size distribution of h-TAc, Figure S1: Particle size distribution of h-TAc, Figure S2: PL spectra of MAPbI_3_ layers incorporated with various h-TAc amounts, Figure S3: PL spectra of PVSC, PVSC-meso, and PVSC-hTAc85, Figure S4: Photocurrent density-voltage curves of PVSC-meso, Figure S5: SEM images of MAPbI_3_ layers incorporated with various TiO_2_ of 0.85-wt%: (a,b) h-TAc, (c,d) P25, (e,f) ST01, (g,f) 18NR-T, Figure S6: PL spectra of MAPbI_3_ layers incorporated with different kinds of TiO_2_, Figure S7: Cross-sectional SEM image of PVSC-18NRT: Figure S8: FTIR spectra of h-TAc and P25.
