# Supplementary material for: MAPbI3 Incorporated with Carboxyl Group Chelated Titania for Planar Perovskite Solar Cells in Low-Temperature Process
Source: Nanomaterials (Basel). 2019 Jun 23;9(6):908. doi: 10.3390/nano9060908 (PMC6631502; doi:10.3390/nano9060908)
Supplement: Supplementary file 1 [file nanomaterials-09-00908-s001.pdf]

Supporting Information

MAPbI$_3$ incorporated with carboxyl groups chelated titania for planar perovskite solar cells in low-temperature process

Pei-Shan Li[a], Rathinam Balamurugan[a], Bo-Tau Liu[a,*], Rong-Ho Lee[b,*], and Hsueh-Tao Chou[a]

[a]Department of Chemical and Materials Engineering, National Yunlin University of Science and Technology, Yunlin 64002, Taiwan, ROC

[b]Department of Chemical Engineering, National Chung Hsing University, Taichung 40227, Taiwan, ROC

*Corresponding author E-mail address: liubo@yuntech.edu.tw (B.-T. Liu); rhl@dragon.nchu.edu.tw (R.-H. Lee).

List of contents

Synthesis of CH$_3$NH$_3$I

Preparation of h-TAc

Table S1. Particle size distribution of h-TAc.

Fig. S1. Particle size distribution of h-TAc.

Fig. S2. PL spectra of MAPbI$_3$ layers incorporated with various h-TAc amounts.

Fig. S3. PL spectra of PVSC, PVSC-meso, and PVSC-hTAc85.

Fig. S4. Photocurrent density-voltage curves of PVSC-meso.

Fig. S5. SEM images of MAPbI$_3$ layers incorporated with various TiO$_2$ of 0.85-wt%: (a, b) h-TAc, (c, d) P25, (e, f) ST01, (g, f) 18NR-T.

Fig. S6. PL spectra of MAPbI$_3$ layers incorporated with different kinds of TiO$_2$.

Fig. S7. Cross-sectional SEM image of PVSC-18NRT.

Fig. S8. FTIR spectra of h-TAc and P25.

Synthesis of CH_3NH_3I

20 ml of hydroiodic acid was slowly dropped in to 50 ml of CH_3NH_2 and stirred for 2 h at 0 ° C. Then the solvent was removed by rotary evaporator. The as-prepared powders was dissolved in ethanol and re-crystalized with diethyl ether. The process was repeated three times, resulting in CH_3NH_3I.

Preparation of h-TAc

Titanium (IV) isopropoxide (12 g) was mixed with acetic acid (2.4 g). After 10 min, the solution was slowly dropped in to 200g of deionized water. After 12-h stirring at room temperature, the solution was added with 2 g of nitric acid and heated at 80 ℃ for 75 min, resulted a stable light-blue suspension of TiO_2 (TAc). The as-prepared TAc solution (100g) was placed in a 225ml autoclave and heated at 250 ° C for 12 hours. The resulting solid was washed three times with deionized water through centrifugation (12000 rpm for 30 min). The purified solid was re-dispersed into DMF, resulting in h-TAc solution (5.8 wt%).

Table S1. Particle size distribution of h-TAc.

	Distribution Result （nm）	D10 （nm）	D50 （nm）	D90 （nm）
h-TAc	82.3	55.5	74.7	115.1

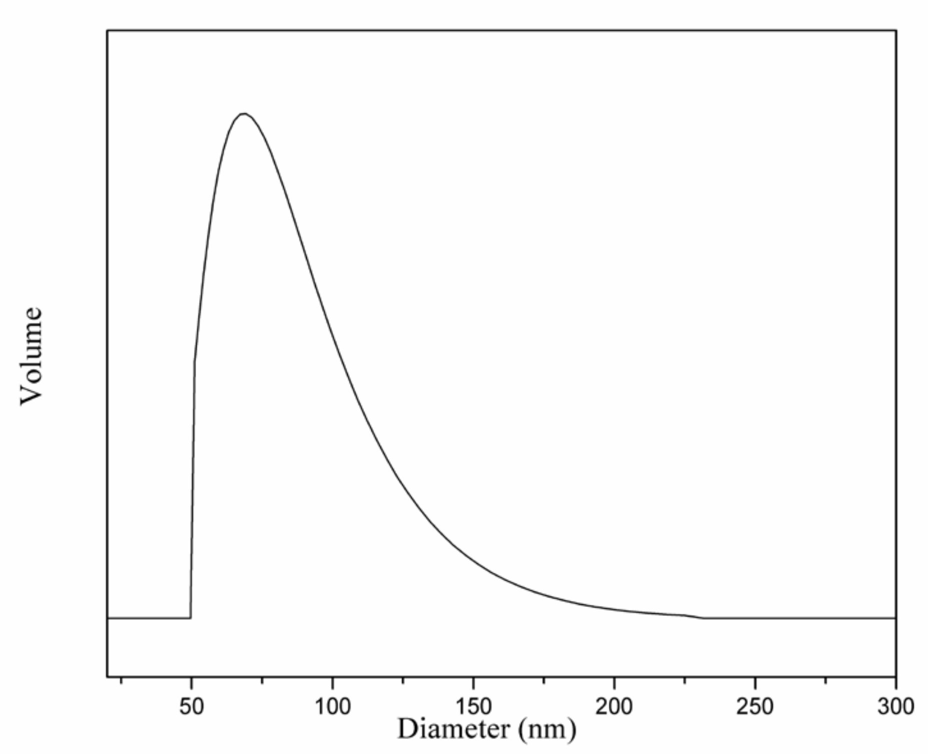

Fig. S1. Particle size distribution of h-TAc.

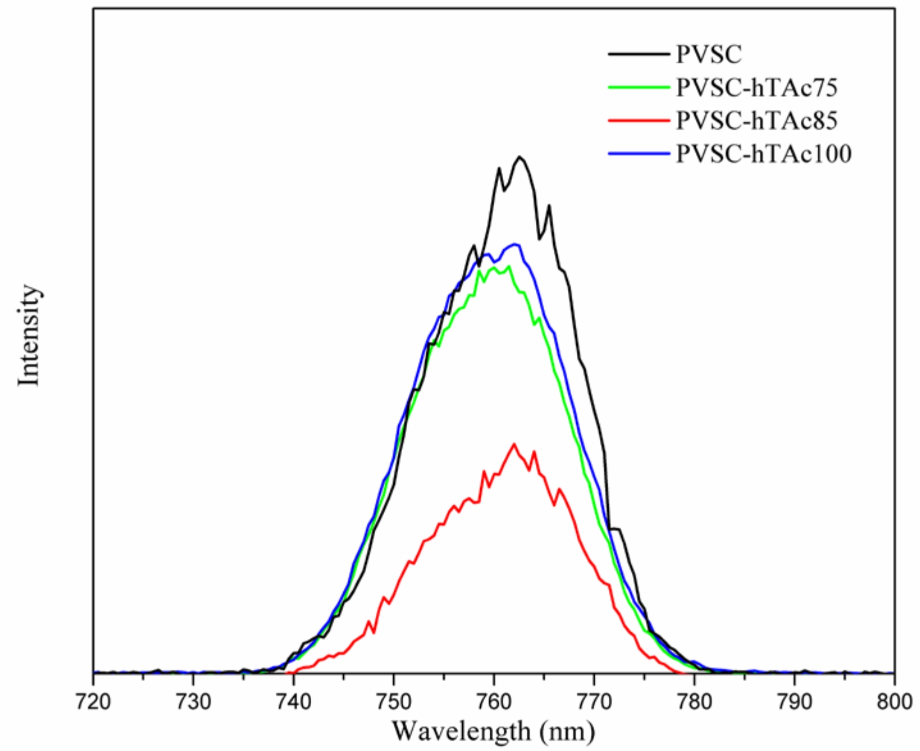

Fig. S2. PL spectra of $MAPbI_3$ layers incorporated with various h-TAc amounts.

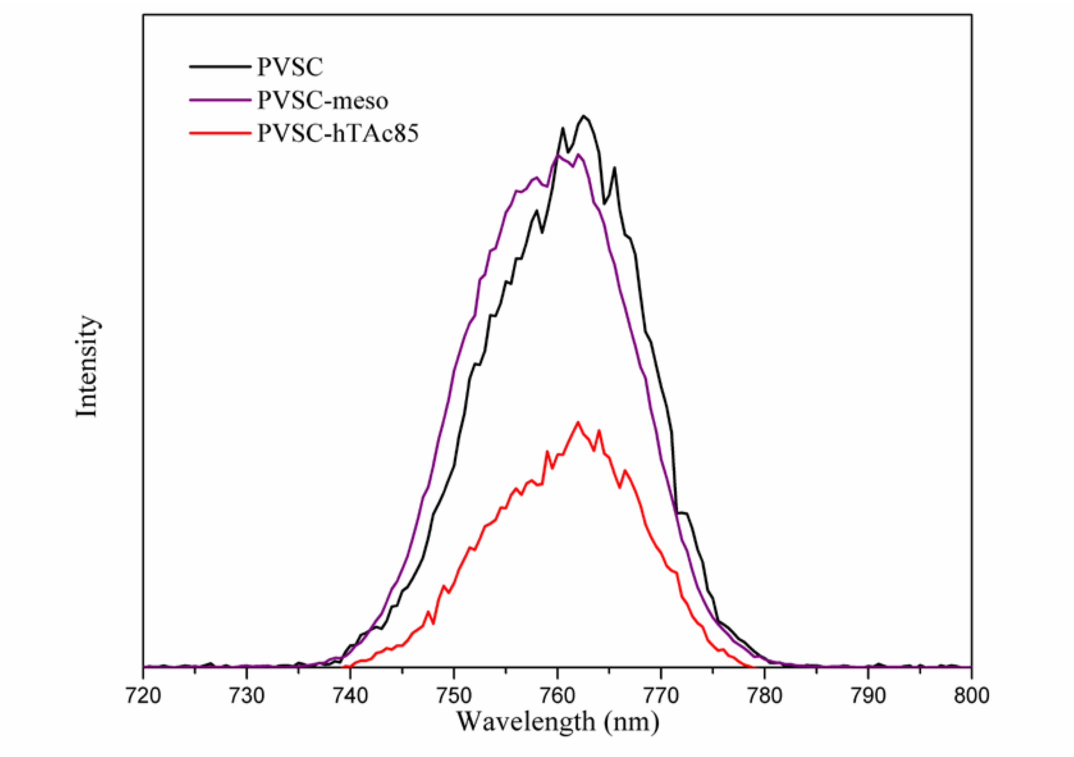

Fig. S3. PL spectra of PVSC, PVSC-meso, and PVSC-hTAc85.

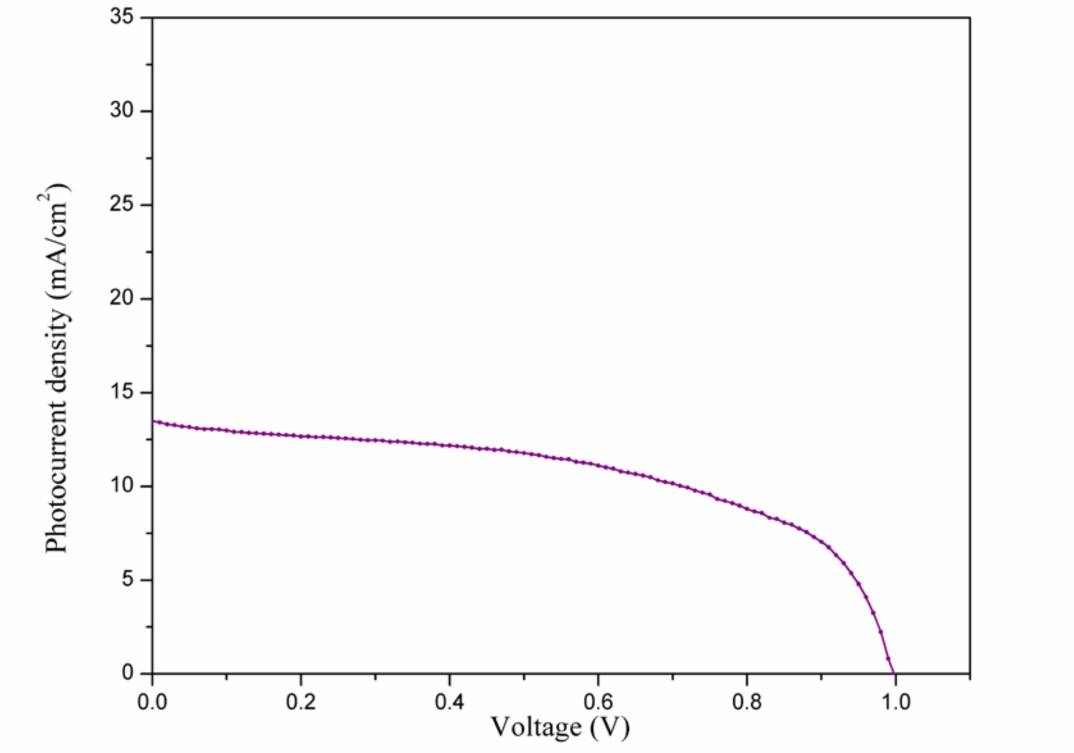

Fig. S4. Photocurrent density-voltage curves of PVSC-meso.

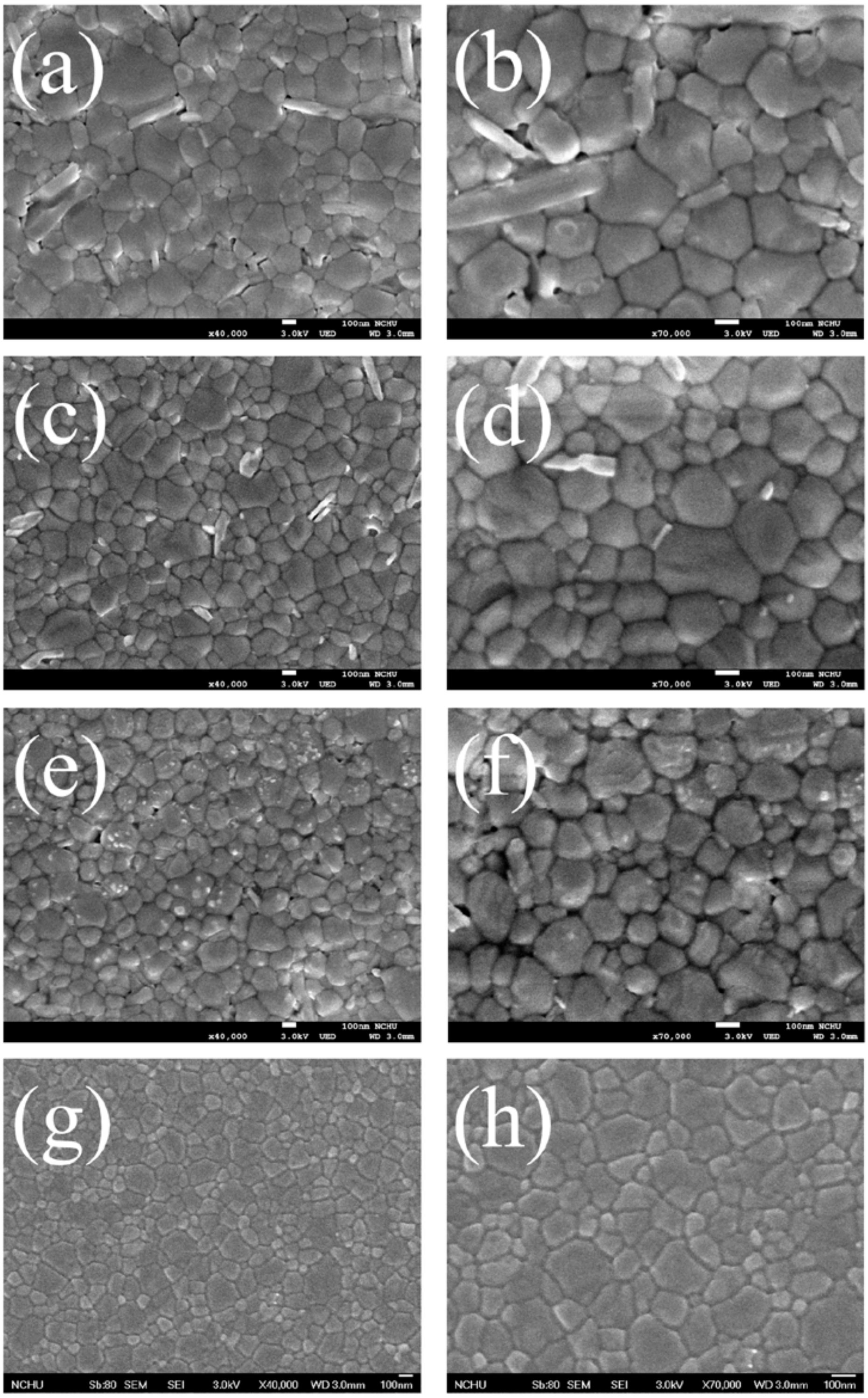

Fig. S5. SEM images of $MAPbI_3$ layers incorporated with various TiO_2 of 0.85-wt%: (a, b) h-TAc, (c, d) P25, (e, f) ST01, (g, f) 18NR-T.

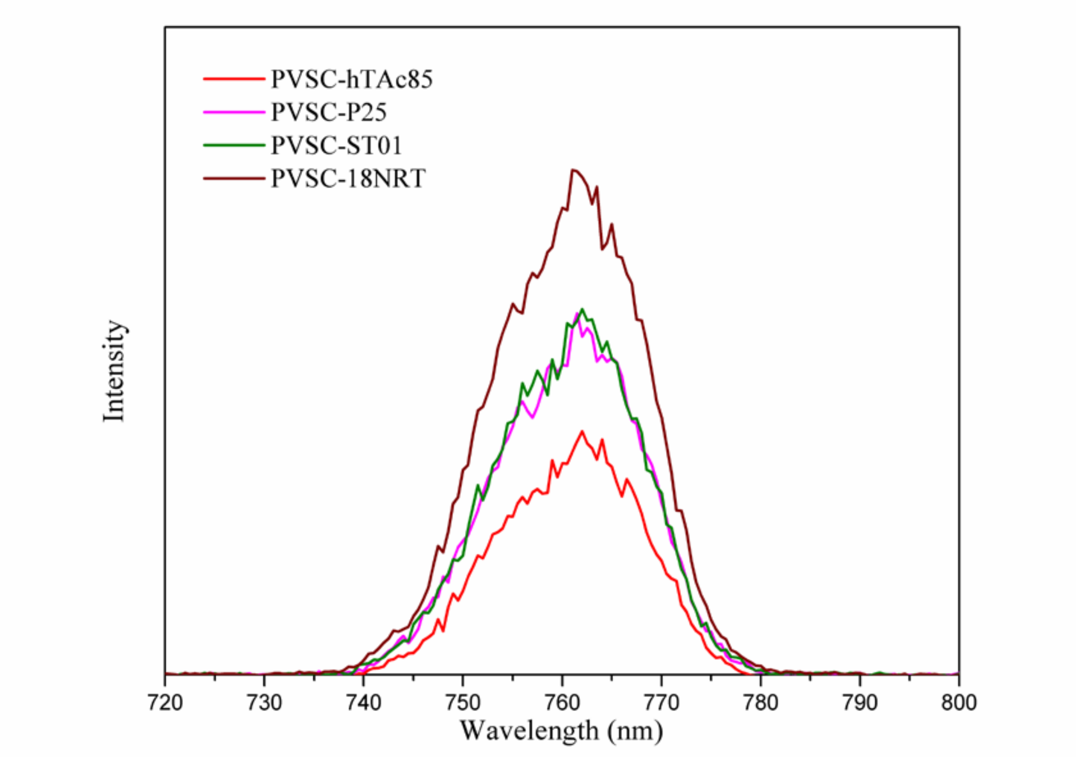

Fig. S6. PL spectra of $MAPbI_3$ layers incorporated with different kinds of TiO_2.

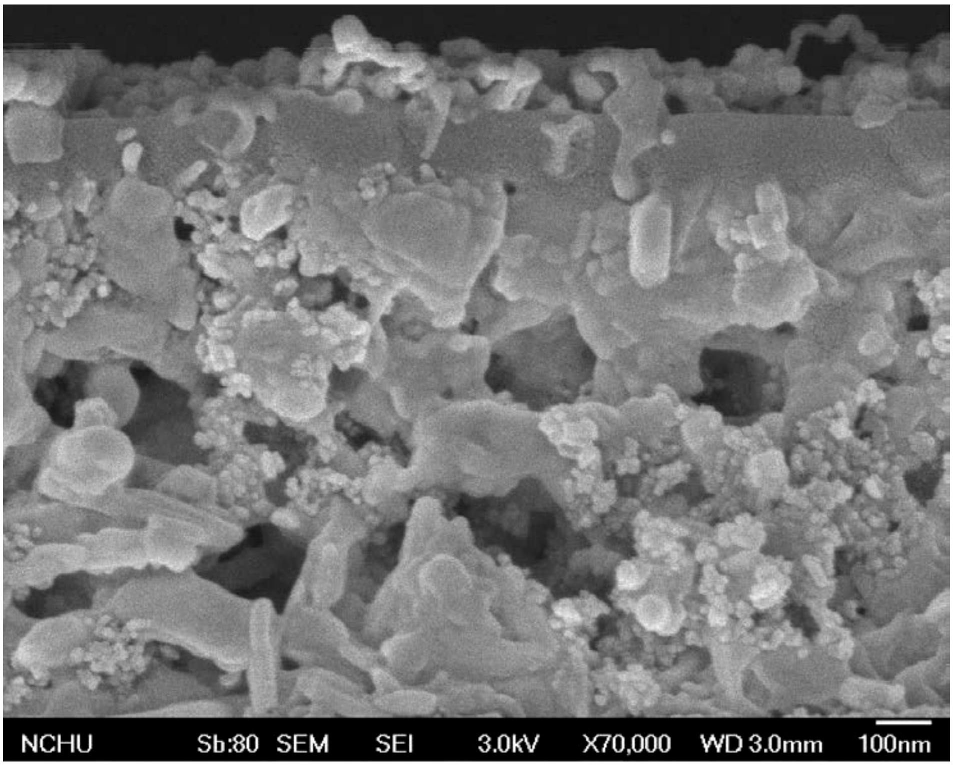

Fig. S7. Cross-sectional SEM image of PVSC-18NRT.

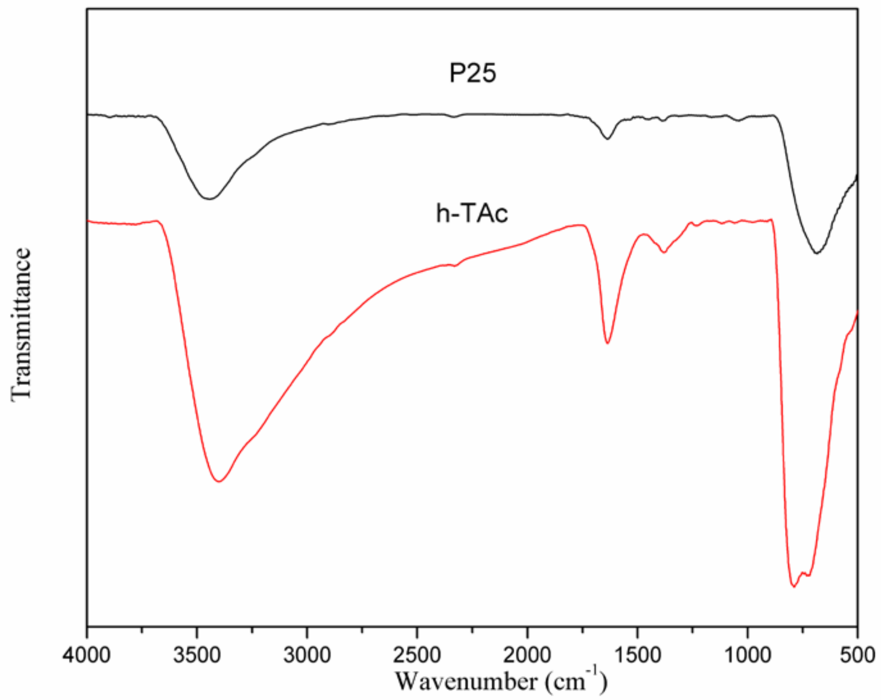

Fig. S8. FTIR spectra of h-TAc and P25.
